# Clinicopathological Findings and Comprehensive Review of Buschke–Lowenstein Tumors Based on a Case Study

**DOI:** 10.3390/jpm14080887

**Published:** 2024-08-22

**Authors:** Andreea Grosu-Bularda, Cristian-Sorin Hariga, Catalina-Stefania Dumitru, Nicolae Calcaianu, Cosmin-Antoniu Creanga, Valentin Enache, Silvia-Elena Tache, Eliza-Maria Bordeanu-Diaconescu, Vladut-Alin Ratoiu, Razvan-Nicolae Teodoreanu, Ioan Lascar

**Affiliations:** 1 Department 11, Discipline Plastic and Reconstructive Surgery, Bucharest Clinical Emergency Hospital, University of Medicine and Pharmacy Carol Davila, 050474 Bucharest, Romania; andreea.grosu-bularda@umfcd.ro (A.G.-B.);; 2 Clinic of Plastic Surgery and Reconstructive Microsurgery, Clinical Emergency Hospital of Bucharest, 014461 Bucharest, Romania; 3 Department of Anatomical Pathology, Clinical Emergency Hospital of Bucharest, 014461 Bucharest, Romania

**Keywords:** giant condyloma acuminatum, Buschke–Loewenstein tumor, human papillomavirus, surgical treatment, histopathological findings

## Abstract

The Buschke–Löwenstein tumor (BLT), also known as giant condyloma acuminatum, is a rare, exophytic tumor, arising from pre-existing warty lesions associated with human papillomavirus (HPV) infection, particularly strains 6 and 11, which are considered to have low oncogenic potential. BLT presents as a large, cauliflower-like growth typically affecting the penis, vulva, vagina, perineum, scrotum, anus, and perianal area. Despite being a benign lesion, BLT is locally aggressive with a high recurrence rate, and can potentially undergo malignant transformation into squamous cell carcinoma, contributing to an overall mortality rate of 20–30%. The primary treatment is complete surgical excision with wide margins, frequently requiring complex reconstructive techniques for defect coverage. We report on a 68-year-old patient, with multiple comorbidities, who presented with a two-year history of a large exophytic tumor in the genital region, affecting the penis, along with progressive erectile dysfunction and urinary problems. The tumor was surgically excised with oncological safety margins, and reconstruction was performed using advancement and rotation flaps from the scrotum and intact penile skin. Histopathological examination confirmed the diagnosis of Giant Condyloma (Buschke–Löwenstein tumor), showing acanthosis, papillomatosis, parakeratosis, and koilocytic cell collections, with positive immunohistochemical staining for p16, p63, and ki67. Postoperatively, the patient had a good clinical outcome and a complete surgical cure. This case highlights the critical need for timely intervention and comprehensive management strategies in treating giant condyloma, given its potential for local invasion and substantial impacts on patient quality of life. Early diagnosis and thorough surgical excision are crucial for effective management and to reduce the high recurrence, morbidity and malignant transformation risk associated with this condition.

## 1. Introduction

The Buschke–Löwenstein tumor (BLT), also known as giant condyloma acuminatum, was first described in 1925 by dermatologists Abraham Buschke and Ludwig Löwenstein, after whom the tumor is named. This initial case report followed Buschke’s earlier description of two venereal invasive condylomas of the penis in 1896 [1,2,3]. 

BLT is a rare condition, with an estimated prevalence of 0.1%, that develops from pre-existing warty lesions linked to human papillomavirus (HPV) infection, particularly strains 6 and 11, which are considered to have a low oncogenic risk [4,5,6,7].

Over time, most people are exposed to HPV infection, making it the most common sexually transmitted disease worldwide. HPVs are small, double-stranded DNA viruses with a tropism for stratified squamous epithelial tissues [8,9,10,11]. Based on their DNA structure, they are classified into five groups: α-HPV, β-HPV, γ-HPV, μ-HPV, and ν-HPV [8,12]. The α-HPV viruses, which have a tropism for cutaneous and mucosal tissues, include subtypes 6 and 11, the main ones involved in the pathogenesis of Buschke–Löwenstein tumor (BLT) [8].

In most cases (90%), the host’s immune response effectively controls the infection, eliminating the viral load. The infection becomes chronic in only 10% of cases [9]. The E6 oncoprotein inhibits the transcription of the p53 protein, a known tumor suppressor, which leads to an increase in R-loop levels [13,14]. R-loops are RNA–DNA hybrids that play a role in transcription regulation; their chaotic formation results in genomic instability and is normally controlled by the RNase H1 enzyme, which degrades R-loops. A significant number of R-loops have been discovered in HPV-infected cells, along with a decrease in the amount of RNase H1 enzyme, leading to the reduced expression of DNA repair genes such as ATR and FANCD2. Conversely, the high expression of RNase H1 has been associated with the inhibition of viral transcription and replication, as well as the stimulation of the innate immune response [13]. HPV viruses can replicate without causing cytolysis, leading to a silent response to the infection without triggering an immune response. The E5 oncoprotein reduces the expression of HLA class I, while E6 and E7 induce an immune tolerance process. These factors contribute to the long-term persistence of HPV infection and promote the progression into cancer [9]. HPV viruses are responsible for approximately 5% of all cancers, according to WHO [15]. Extensive cutaneous manifestations of HPV infections, such as those seen in Buschke–Löwenstein tumor (BLT), have been primarily described in patients with immune system disorders, such as those with HIV infection [8].

Buschke–Löwenstein tumors are more prevalent in males than females, with a male-to-female ratio of 2.7:1. They most commonly occur between the fourth and sixth decades of life, particularly affecting uncircumcised males under 50 years old, with higher incidence rates among homosexual and bisexual men, and they are rarely seen in children. No racial predispositions have been identified for this tumor [3,4,16,17].

BLT presents as a large, exophytic tumor with a cauliflower-like appearance. It typically affects the penis, vulva or vagina, perineum, scrotum, anus, and perianal area, and is characterized by its slow growth [1,18]. 

Although BLT is a benign lesion, it is locally aggressive, and there is a possibility of malignant transformation. BLT has a recurrence rate of up to 67% and is locally destructive. It does not involve lymph nodes, nor does it invade vascular or neural structures, and distant metastases have not been documented. Malignant transformation into squamous cell carcinoma (SCC) has been reported in 30–56% of cases, with an overall mortality rate of 20–30% [1,19,20,21]. 

Buschke–Löwenstein tumors are typically diagnosed through a detailed clinical history and visual examination of the lesions. Additional diagnostic support can be provided by DNA detection assays such as polymerase chain reaction (PCR), which can confirm the diagnosis and allow for gene typing. If there is concern about malignant transformation, a biopsy of the lesion should be performed as part of the evaluation. Signs that may indicate malignant transformation in BLT include bleeding, irregular pigmentation, ulceration, and lesions with palpable tissue infiltration [3,5,22,23].

The main therapeutic approach for Buschke–Löwenstein tumors is represented by complete surgical excision with wide margins, which aims to remove the tumor entirely due to its high recurrence rate, often requiring complex reconstructive procedures for defect coverage [1,5,22,23,24,25]. Other therapeutic approaches include CO_2_ laser ablation, topical agents like podophyllin and imiquimod, and systemic therapies such as interferon. Radiotherapy is also an option, particularly for inoperable or recurrent cases, though it is controversial due to the potential risk of malignant transformation. The management of extensive or recurrent BLT remains a subject of ongoing debate, with no consensus on the most effective long-term strategy [1,3,4,5,22,23,24,25,26].

Buschke–Löwenstein tumors represent a controversial subject, primarily due to limited clinical trials and high-level evidence, especially concerning screening and prevention strategies. Currently, around the world, there are no generally established guidelines for the large-scale vaccination of males or screening practices for squamous cell carcinoma or high-grade squamous intraepithelial lesions. This lack of consensus highlights the need for more research to develop effective prevention and management strategies for BLT [27,28].

## 2. Case Presentation

In conducting and reporting this study, we adhered to the ethical principles outlined in the Declaration of Helsinki. Written informed consent was obtained from the patient for scientific purposes and accompanying images, according to our hospital protocols.

A 68-year-old patient presented to our clinic with a complaint of a tumor in the genital region that had been developing over the past two years without any therapeutic intervention. The patient initially presented to the territorial dermatology service, where the diagnosis of condylomatosis was made based on a biopsy, establishing the HPV etiology. He was referred to Plastic Surgery, as the extent of the lesions would require a reconstructive procedure. The patient had a significant medical history, including arterial hypertension, a benign adrenal tumor, sequelae of respiratory tuberculosis, COPD, and depressive disorder. He reported progressive erectile dysfunction and urinary problems associated with the tumor. 

**Figure 1:**
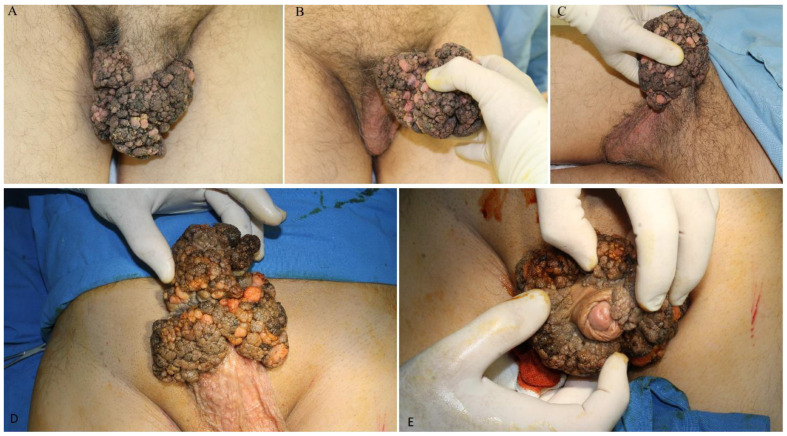
(**A**–**C**) Preoperative frontal and lateral views of penile condylomatosis. (**D**,**E**) Despite the tumor extension, there is no involvement of the penile gland, partial foreskin, or scrotal skin.

At clinical examination, a large exophytic tumor with a cauliflower-like appearance was noted in the genital region, spreading from the penis to the anterior surface of the scrotum (Figure 1A–C), extending in surface for 15 cm × 10 cm and having a projection between 2.5–3.5 cm, with a narrower implantation base compared to the maximum surface area extension and maintaining mobility of the deep planes. The retraction of the foreskin allowed visualization of the external urinary meatus and partial exposure of the glans penis, without any modification at these levels (Figure 1D,E). The urological examination ruled out invasion at the urethral level, with the tumor extending only to the penoscrotal skin. No locoregional lymphadenopathy was detected. Given the clinical presentation, further investigations were deemed necessary to determine the nature of the tumor and plan appropriate management.

Initial laboratory investigations revealed normal leukocyte counts and the absence of elevated inflammatory markers. Viral screening for HIV, hepatitis B, and hepatitis C was negative. Chest X-ray showed no acute pulmonary lesions but demonstrated bilateral apical micronodular and nodular opacities with a sequelae-like appearance and emphysematous changes in the right lung. There was no microbial growth found in the tests performed. 

Under these circumstances, a therapeutic surgical procedure was undertaken, the surgical team being led by the senior author. Since the investigations revealed a significant local extension of the lesion, without clinical signs of deep invasion, the therapeutic indication is for radical excision of the tumor, which, in our opinion, is the treatment method of choice.

**Figure 2:**
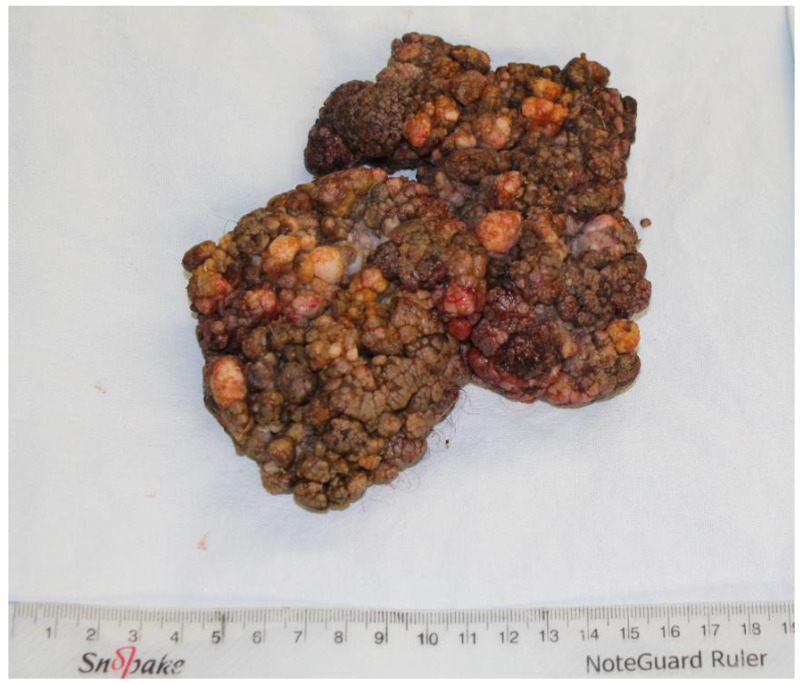
Completely resected tumor mass.

**Figure 3:**
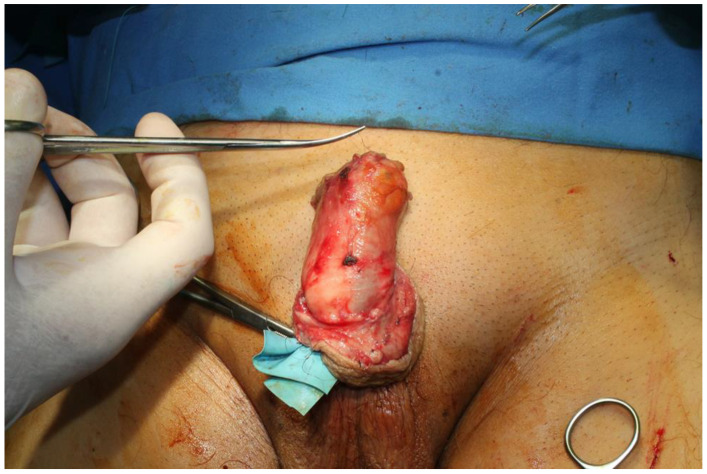
The residual defect following penile tumor resection.

**Figure 4:**
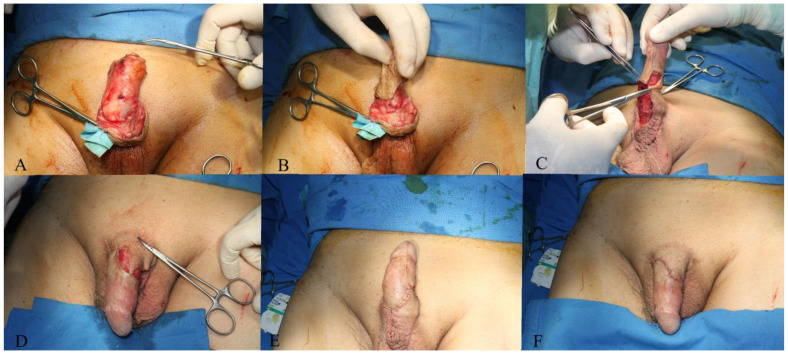
(**A**–**D**) The penile shaft is reconstructed using scrotal and foreskin advancement and rotational flaps. (**E**,**F**) The outcome of penile reconstruction following the inset of flaps.

The tumor was completely excised, ensuring adherence to oncological safety margins and extending into macroscopically healthy tissue, with a margin of 6 mm (Figure 2). The excised tumor was sent for histopathological examination. After tumor excision, hemostasis was performed and the defect was assessed, resulting in a circumferential defect at the level of the penile shaft, with a maximum longitudinal defect of 9 cm on the ventral side and 4.5 cm on the dorsal side of the penis. After the undermining of the adjacent remaining skin flaps and their approximation, the resultant defect measured approximately 6 cm by 4 cm, situated on the ventral and partially dorsal surfaces of the penis (Figure 3). The reconstruction of this defect was achieved using advancement and rotation flaps, sourced proximally from the scrotum and distally from the remaining intact foreskin and dorsal penile tissues. The choice of utilizing local and regional tissue for reconstruction was made to ensure a like-to-like restoration, aiming to achieve optimal functional outcomes in this anatomically critical area (Figure 4). 

**Figure 5:**
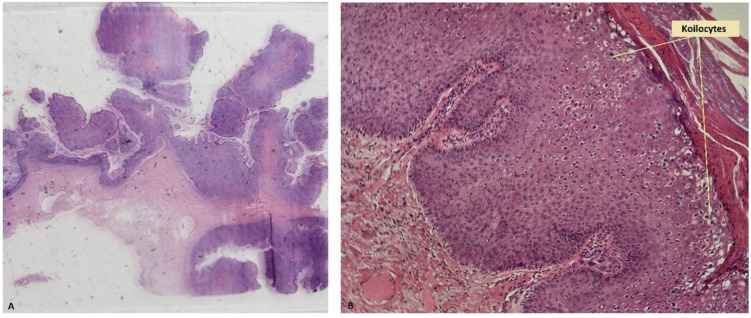
(**A**) Hematoxylin and eosin staining at 25× magnification, revealing exuberant vegetative appearance with multiple pseudopapillary structures, without invasive characteristics on section. (**B**) Hematoxylin and eosin staining at 100× magnification, revealing acanthosis, papillomatosis, and parakeratosis, with areas of the epidermis containing collections of koilocyte-type cells, occasionally involving the entire thickness of the epidermis. No atypical cells are observed.

**Figure 6:**
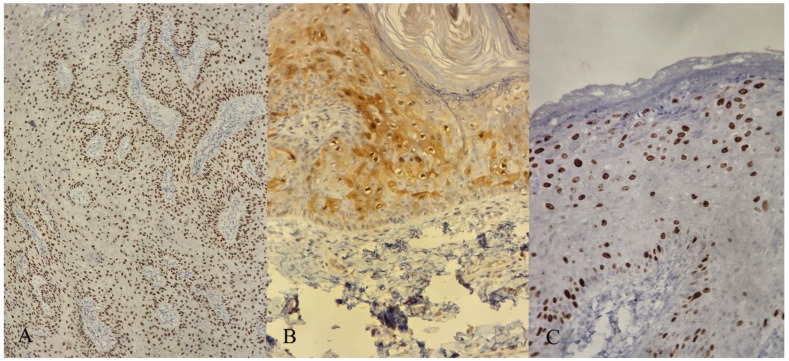
Immunohistochemistry showing: (**A**) Epithelial cells’ diffuse nuclear staining for p63 (100× magnification). (**B**) Positive nuclear and cytoplasmic staining for p16 was observed in 60% of the cells (200× magnification). (**C**) Ki-67 is positive in approximately 60% of basal cells and in regions of the middle and upper layers of the epidermis (200× magnification).

Histopathological examination of the excised tumor revealed macroscopic features consistent with a 15 cm × 10 cm exophytic growth exhibiting pseudopapillary structures. Microscopic examination showed skin fragments with acanthosis, papillomatosis, parakeratosis, and koilocytic cell collections throughout the epidermis (Figure 5). Immunohistochemical analysis demonstrated positive staining for p16 and p63 in the epidermis, and ki67 positivity in the basal and upper layers of the epidermis (Figure 6).

Based on the histological and immunohistochemical findings, a diagnosis of giant condyloma (Buschke–Löwenstein tumor) was established.

Postoperatively, the patient received multimodal analgesia, proton pump inhibitors for gastric ulcer prophylaxis, antibiotic prophylaxis, and thromboprophylaxis with low-molecular-weight heparin. Wound care included Betadine irrigation, topical antiseptics, and sterile dressing changes. Throughout hospitalization, the patient maintained stable hemodynamics and respiratory function, and remained afebrile and oriented.

With a favorable evolution, the patient was discharged from the hospital on the 5th postoperative day and returned regularly for follow-up and dressing changes. He presented with progressive wound healing and stable vital signs. The sutures were removed at 14 days, and the patient resumed local hygiene. The patient was referred to another service for HPV PCR genotyping and was informed about the risk of developing new lesions, but did not return with the investigation results. In evolution, the patient remains free of residual disease, achieving a complete surgical cure, with a favorable local and functional outcome at one-year follow-up. 

## 3. Discussion and Literature Review

Buschke–Löwenstein tumors represent a rare condition attributed to the sexually transmitted HPV infection, with limited case reports and studies available in the global medical literature [1,8,29].

Human papillomavirus (HPV) infection is highly prevalent worldwide and is considered the most common sexually transmitted disease along with Chlamydia trachomatis [8,30]. 

The incidence of HPV infection increases proportionally with the number of sexual partners, and it is higher among populations with an early onset of sexual activity. Other populations at increased risk for HPV infection include sex workers, men who have sex with men, bisexual individuals, and those with chronic genital infections or poor hygiene [8,22,31]. In immunocompetent patients, most infections with low-risk HPV strains are either asymptomatic or self-limiting [8,32]. It is estimated that every individual encounters at least one HPV strain during their lifetime, with the majority of infections being asymptomatic; genital warts occur in only 2% of cases [22]. However, in immunocompromised patients, the same infection can have devastating effects, leading to extensive lesions that may exhibit malignant characteristics [8]. CD4+ T lymphocytes play a crucial role in the immune response against HPV, as evidenced by the increased incidence of extensive, dysplastic lesions in patients with HIV infection, those undergoing immunosuppressive treatments, or individuals with congenital immune system errors, such as mutations in the GATA2, DOCK8, and CXCR4 genes [8,33]. Most studies associate Buschke–Löwenstein tumors with low-risk human papillomavirus (HPV) types 6 and 11. The genomes of these HPV strains encode DNA sequences that translate into E (early) 6 and E7 proteins. These proteins bind to the tumor suppressor protein p53, accelerating its degradation. This process immortalizes epithelial cells, allowing them to replicate unchecked and accumulate DNA mutations, leading to chromosomal instability and, ultimately, abnormal growth manifesting as BLT and also having malignant transformation potential [3,34,35,36].

Several causative risk factors for Buschke–Löwenstein giant condylomas have been identified. These include long-standing phimosis, inadequate penile hygiene (particularly in uncircumcised males), immunosuppression (such as from HIV or immunosuppressive medications), chemotherapy, prolonged irritation (e.g., from ulcerative colitis or perianal fistulas), diabetes, smoking, substance abuse including alcoholics, pregnancy, and poor socioeconomic status [3,17,37]. Most patients diagnosed with this pathology have a degree of congenital or acquired immunodeficiency, complicating treatment due to the increased rate of postoperative infections and the slower healing process in these patients [8].

The hallmark of BLT is its aggressive behavior, characterized by local destructiveness and an extreme invasive capacity, despite the absence of histological features of malignancy [1,16,38]. Macroscopically, it presents as an exophytic, cauliflower-like formation with a tendency for impressive, destructive growth, locally mimicking a malignancy and exhibiting a very high recurrence rate (Figure 1) [1,21]. The majority of Buschke–Löwenstein tumors are located on the penis (81–94%) in men and the vulva (90%) in women. Other common sites include the anorectal region and the urethra in men, but it may affect the perineum, scrotum, vagina, and even the bladder in severe cases [1,22].

Buschke–Löwenstein tumor (BLT) accounts for 5% to 24% of reported penile tumors in the U.S. [3]. The differential diagnosis of BLT is critical because its appearance can be similar to other benign and malignant penile conditions. Although clinical features and imaging studies, such as CT or MRI, are highly suggestive of BLT, a definitive diagnosis can only be made through histopathological evaluation. Sometimes the appearance of the tumors can be nonspecific, as indurated tumor mass may include ulcerations or nodular lesions [3,22]. Penile or vulvar tumors, including squamous cell carcinoma, verrucous carcinoma, basal cell carcinoma, melanoma, seborrheic keratosis and lymphangioma circumscriptum, and other malignancies (including very rare tumors such as Kaposi sarcomas, solitary fibrous tumors and other types of sarcoma) can sometimes be misdiagnosed as giant condylomas [39,40,41,42,43,44,45,46].

Key differentials include verrucous carcinoma, squamous cell carcinoma (SCC), and condyloma acuminatum (genital warts) [3,47].

Verrucous carcinoma is a low-grade, well-differentiated variant of SCC, which, like BLT, presents with a verrucous or wart-like appearance. However, verrucous carcinoma tends to invade deeper tissue and can be more aggressive, whereas BLT typically exhibits a locally invasive growth pattern without metastasizing [3,39,47].

Squamous cell carcinoma is another important differential, as it is a more aggressive malignancy compared to BLT. SCC can present with ulceration and a more irregular, nodular surface, unlike the exophytic, papillomatous growth of BLT. Histopathological examination is essential to differentiate SCC, as it often shows atypical keratinocytes with significant nuclear pleomorphism and high mitotic activity, contrasting with the benign appearance of BLT’s epithelial cells [3,5,16,17,48].

Condyloma acuminatum, caused by human papillomavirus (HPV) infection, shares a similar etiology with BLT, but generally presents as smaller, softer, and less aggressive lesions [3,23].

Given the potential for confusion with these other conditions, a thorough clinical examination coupled with a histopathological analysis is essential for accurate diagnosis. The correct identification of BLT is crucial, as its management differs significantly from those of more aggressive tumors like SCC, often requiring surgical excision with a focus on local control rather than systemic treatment [3,5,22].

These misdiagnoses occur due to the overlapping clinical presentations of these conditions. A biopsy followed by histopathological analysis is a crucial step in diagnosis [3,5,22,39].

BLT is characterized microscopically by papillomatosis with severe acanthosis, hyperkeratosis, parakeratosis, and the presence of koilocytes (Figure 5) [8,22]. Koilocytes are cells typically observed in HPV infections, characterized by mature squamous cells with defects in the keratin network, resulting in a perinuclear halo [49]. The nucleus is irregular, hyperchromatic, and eccentrically located [50]. Other suggestive changes may include the presence of giant keratohyalin granules [50]. Within the papillary dermis, a lymphohistiocytic inflammatory infiltrate is typically observed, without disruption or invasion of the basement membrane. This finding is crucial for differentiating BLT from malignant tumors [22].

The optimal treatment for BLT remains a subject of debate due to the absence of a consistent series of patients and a well-defined treatment protocol. Various treatment options are documented in the literature, each yielding different outcomes. Radiotherapy has shown success in some cases, including total regression, but there is also evidence of anaplastic transformation and the extensive emergence of new condylomas following its use. The use of radiotherapy is controversial due to the lack of clear recommendations regarding dose and fractions. Other treatment modalities often employed include cryosurgery, CO_2_ laser surgery, podophyllin, intralesional bleomycin, 13-cis retinoic acid, systemic chemotherapy, topical 5-FU, imiquimod, and interferon-α. However, these methods have not demonstrated consistent effectiveness, particularly for large lesions. The gold standard treatment for BLT consists of wide excision with sufficient margins around the tumor. Despite this approach, the risk of recurrence remains high [1,22,38,51,52,53,54].

While some authors suggest using a full-thickness skin graft to cover the resultant defect, others support allowing the wound to heal by secondary intention. This latter approach is favored because HPV requires the presence of stratified squamous epithelial tissue to multiply. Fibrotic tissue formed during secondary intention healing is less likely to undergo local recurrence [22]. This approach may be taken into consideration for limited defects. In the therapeutic management of extensive defects resulting from the excision of giant condylomas, more complex reconstructive methods are often required. These typically involve the use of regional flaps or free tissue transfer [5,26,51,55,56]. The case should be managed by a multidisciplinary team to establish an appropriate therapeutic approach, which may include the potential need for colostomy or nephrostomy placement [22].

Since giant condyloma affects immunocompromised patients, including those with HIV or in the AIDS stage, there are specific therapeutic implications and precautions that must be considered to avoid complications. Infectious complications due to bacterial superinfections are frequently encountered in these patients, sometimes constituting the reason for emergency hospital admission [56].

Atkinson et al. detailed the clinical management of two HIV-positive patients diagnosed with giant condylomas complicated by urinary outflow obstruction and sepsis [57].

Radovanovic et al. reported the case of a 63-year-old male with a three-year history of a perianal mass, diagnosed as BLT. He delayed seeking help due to embarrassment, presenting with pain, discharge, bleeding, and a foul odor. Extensive surgical excision and fistula removal were performed, with the wound left open for secondary healing and a colostomy to prevent fecal contamination. Despite sustained multimodal treatment, the patient’s condition deteriorated, and he died from septic complications 11 months after the initial diagnosis [52].

The presence of perianal abscesses and fistulas predisposes one to severe systemic complications, often involving highly virulent and drug-resistant bacteria like those pertaining to the ESKAPE group. Such pathogens can be encountered in immunosuppressed patients or those with multiple comorbidities who have required repeated hospitalizations, as they tend to contract infections with aggressive and multi-resistant bacteria, leading to high morbidity and mortality rates [58,59,60].

The presence of an extensive infection necessitates an aggressive surgical approach, involving repeated debridements, meticulous daily wound care, and systemic antibiotic therapy. Treatment should begin empirically, with broad-spectrum antibiotics followed by targeted antibiotics based on the results of antibiograms. This aggressive management strategy is crucial for controlling the infection and promoting effective healing. If a biopsy is performed and there is no evidence of malignant transformation, the use of Negative Pressure Wound Therapy (NPWT) can be beneficial in the temporary management of defects resulting from the resection of superinfected condylomas or in association with a skin grafting procedure. The innovative combination of NPWT-assisted dressings, enhanced with a silver nanoparticle sheet, led to significant improvements in graft integration and a reduction in systemic inflammation, having also an important antimicrobial effect [56,61,62,63].

The malignant transformation of BLT occurs in up to 50% of cases, with key clinical indicators being local bleeding or the palpation of enlarged regional lymph nodes [1,22]. Persistent infection with papillomavirus for more than 6 months increases the risk of oncogenic transformation in infected cells. In cases of malignant transformation, thorough investigations are necessary to determine the extent of invasion into adjacent tissues [22,33].

Additionally, a more rapid progression of Buschke–Löwenstein tumors (BLTs) to squamous cell carcinomas (SCC) has been observed in HIV-infected patients, with the extent of genital lesions directly correlating with a decrease in CD4+ T lymphocyte count and regression upon initiation of antiretroviral therapy [8].

Several public health strategies have been implemented to reduce the transmission of HIV and HPV and to mitigate the effects of coinfection. Among these, universal HPV vaccination stands out as the most effective preventive measure to lower the incidence of HPV-related diseases [64].

The HPV vaccine is currently one of the most effective methods for preventing this sexually transmitted infection [9,10,15,65]. All vaccine types include recombinant L1 proteins that generate virus-like particles and stimulate the immune system to produce antibodies against HPV [15]. Currently, there are six approved HPV vaccines, three of which are effective against types 6 and 11, which are involved in Buschke–Löwenstein tumor (two quadrivalent and one nonavalent) [9,65]. The WHO advises prioritizing HPV vaccination for girls aged 9 to 14 years who have not yet become sexually active and also recommends vaccination for women over 15 years and men. It is projected that reaching an 80% vaccination coverage in both sexes could lead to the complete eradication of HPV types 6, 11, 16, and 18 through herd immunity [9]. Recent studies have shown that HPV vaccination significantly reduces the mortality rate associated with this viral infection, both in women and men [66].

Due to the high risk of recurrence, close and long-term follow-up is essential in Buschke-Lowenstein tumors. Follow-up strategies should include regular clinical examinations and imaging, particularly in the first few years post-treatment, as this is the period with the highest risk of recurrence. Patients should be monitored for any signs of local recurrence, new lesions, or complications related to the tumor’s invasive nature. Long-term follow-up is critical to ensure the early detection of any recurrences or potential malignant transformation, thereby improving overall outcomes [4,6,8,26].

We chose to present our clinical case due to the rarity of Buschke-Lowenstein tumors, particularly in immunocompetent individuals. Despite not being in the typical at-risk group, our patient likely had predisposing factors due to comorbidities and advanced age. Although the patient had no diagnosed acquired immunodeficiency, such as HIV, his history of tuberculosis and COPD might suggest a weakened immune system, making him more susceptible to severe, atypical HPV infections. Consistent with cases described in the literature, the patient did not seek medical assistance until the disease significantly impacted his daily life, causing urinary difficulties and severe erectile dysfunction. The therapeutic decision focused on surgical treatment, currently considered the gold standard for this pathology, involving wide and complete resection to achieve clear margins both macroscopically and microscopically.

Histopathological examination revealed typical changes for this type of tumor, including significant structural alterations in the epidermis and the presence of koilocytic cells indicative of HPV infection, without invasive features affecting the underlying structures. Although no malignant transformation was detected at the time of surgery, the presence of aggressive markers indicates a higher risk of the further development of squamous cell carcinoma. Immunochemistry in this patient’s case showed Ki-67 positivity in approximately 60% of basal cells, associated with an increased cell division rate and higher tumor aggressiveness, and positive nuclear and cytoplasmic staining for p16 in 60% of the cells, which is associated in the literature with HPV infection [67,68,69,70,71,72]. Unfortunately, the patient did not undergo HPV genotyping, but the histopathological findings confirm the association of the lesions with HPV.

The clinical appearance of a large tumor, combined with the patient’s associated pathology and the mentioned aggressive markers, places this patient in a category that requires close monitoring, given the high recurrence rate of this condition. Periodic clinical examination, involving the entire external genital area, perineum, and perianal region, is crucial to detect any new lesions at an early stage, thus avoiding the risk of developing a recurrence or even a malignant lesion. These preventive and diagnostic measures must also be applied to the patient’s partners.

A limitation of our study was that the patient was referred to another service for HPV PCR genotyping, and was informed about the risk of developing new lesions, but did not follow up with the investigation results. This report underscores the importance of awareness and prompt management of genital tumors, particularly in elderly patients with complex medical histories. Early intervention could potentially improve the quality of life and clinical prognosis for such patients.

## 4. Conclusions

In conclusion, the Buschke–Löwenstein tumor (BLT) is a rare entity resulting from a sexually transmitted disease. Although benign, it is locally aggressive, has a high recurrence rate, and carries the risk of malignant transformation into squamous cell carcinoma (SCC). The effective management of BLT requires a multidisciplinary approach, with the cornerstone of treatment being complete surgical excision with wide margins. This often necessitates complex reconstructive techniques to address the resulting defects and preserve both function and aesthetics.

The presented case highlights the importance of timely intervention and a comprehensive management strategy in treating giant condyloma, particularly given its potential for deep local invasion, significant morbidity, and profound impacts on the patient’s quality of life. Early and accurate diagnosis is crucial, as it allows for prompt and thorough surgical management, which is essential in reducing the risk of recurrence and mitigating the potential for malignant transformation. Additionally, long-term follow-up is imperative due to the high likelihood of recurrence and the potential for delayed malignant transformation, emphasizing the need for ongoing vigilance in the post-treatment period. Extensive HPV vaccination strategies should be promoted to avoid the severe complications generated by this disease.

## Data Availability

Dataset available on request from the authors.
